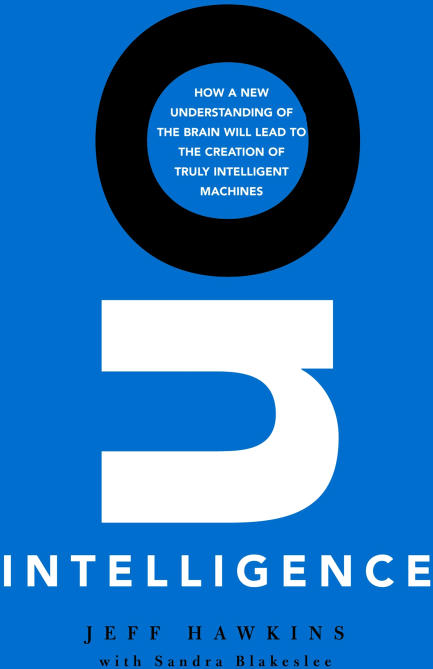# Palmistry

**DOI:** 10.1371/journal.pbio.0020394

**Published:** 2004-11-16

**Authors:** Peter Dayan

## Abstract

A review of "On Intelligence", a book exploring brain function by entrepreneur Jeff Hawkins and science writer Sandra Blakeslee

Is Michael Moore liberal America's Rush Limbaugh? If so, is he filling a much needed, or a much lamented, gap in turning issues that are really cast in pastel shades into Day-Glo relief? In this hale monograph, Jeff Hawkins (rendered by Sandra Blakeslee) plays exactly this role for theoretical neuroscience. As a pastel practitioner myself, but furtively sharing many of Hawkins' prejudices and hunches about computational modelling in neuroscience, I am caught between commendation and consternation.

Hawkins is an engineer, entrepreneur, and scientist who founded and led the companies Palm and then Handspring. He created, against what must have been considerable obstacles, the first widely successful PDA, and continued the development of this platform. He has thus amply earned a bully pulpit. The autobiographical segments of this book detail that, throughout his career, he has been interested in understanding how the brain works, using his substantial knowledge and intuition about the architecture and design of conventional computers as a counterpoint.

More recently, Hawkins has generously put his money where his ideas about mentation dictate, founding the Redwood Neuroscience Institute and also funding various conferences and workshops. The institute is dedicated to ‘studying and promoting biologically accurate mathematical models of memory and cognition.’Despite its youth, the Institute already has attracted notable attention as a centre for theoretical neuroscience. Hawkins' quest, and-depending on which statements of the book you read-its endpoint (‘… a comprehensive theory of how the brain works … describ[ing] what intelligence is and how your brain creates it’) or just its tipping point (‘join me, along with others who take up the challenge’), are the subject here.

There are really three books jostling inside the covers. One is the (highly abbreviated) autobiography. The history of modern computing is very brief and (at least judging by the sales) very glorious, and this story is most entertaining. Don't miss the wonderfully faux naive letter from Hawkins to Gordon Moore asking, in 1980, to set up a research group within Intel devoted to the brain. That Hawkins prospered in clear opposition to accepted wisdom is perhaps one of the key subtexts of the book.

The second, and rather less satisfying, book is about the philosophy of mind and the history of artificial intelligence and neural network approaches to understanding the brain and replicating cognition. With respect to the fields of artificial intelligence and neural nets, the text seems rather to be fighting yesterday's battles. The importance of learning, flexibility in representation and inference, and even decentralisation of control has been more than amply recognised in the inexorable rise of probabilistic approaches in both fields.

With respect to the philosophy of mind, there seems to be something of an enthusiast's disdain for the niceties of philosophical pettifogging, even arguing by assertion. The discussions at the end on creativity and consciousness all seem a bit gossamer. The book is somewhat careless about functionalism, a key doctrine for computational theorists about how brains give rise to minds. According to this doctrine, at least roughly, it is the functional roles of, and functional interactions among, the physical elements of brain that matter, and not their precise physical nature. If you can capture those functional aspects correctly, for instance, in a computer program, then you can (re-)create what's important about mental states. Functionalism licenses a form of inquiry into the computational jobs played by structures in the brain. However, although formally agreeing that ‘there's nothing inherently special or magical about the brain that allows it to be intelligent,’the book slips into statements such as ‘brains and computers do fundamentally different things,’which are, at best, unfortunate shorthand.

The book is a little apt to sneak plausible, but misleading, claims under the radar. Just to give one instance, it compellingly compares a six year old hopping from rock to rock in a streambed with a lumbering robot failing to do the same task. However, this is a bit unfair. One of Hawkins' self-denying ordinances is to consider the cortex pretty much by itself. As aficionados of the cerebellum (an evolutionarily ancient brain region with a special role in the organisation of smooth, precise, well-timed, and task-sensitive motor output) would be quick to point out, the singular role for the cortex in such graceful behaviour is rather questionable.

The third book is what I think is intended to be the real contribution. This contains a (not wholly convincing) attempt to conceptualise the definition of intelligence in terms of prediction rather than behaviour, and then to describe its possible instantiation in the anatomy (and mostly only the anatomy) of the cortex.

## Unsupervised Learning

To situate Hawkins' suggestions, it is instructive to consider current models of how the cerebral cortex represents, and learns to represent, information about the world without being explicitly taught. Being a popular account, the book fairly breezes by these so-called unsupervised learning models (see Hinton and Ghahramani 1997; Rao et al. 2002), in which the neocortex is treated as a general device for finding relationships or structure in its input. The algorithms are called unsupervised since they have to work without detailed information from a teacher or a supervisor about the actual structure in each input. Rather, they must rely on general, statistical characteristics.

First, where does the structure in the inputs come from? For the sake of concreteness, think of the input as being something like movies on a television screen. Movies don't look like white noise, or ‘snow’, because of their statistical structure. For instance, in movies, pixel activities tend to change rather slowly over time, and pixels that are close to each other on the screen tend to have relatively similar activities at any given time. Neither of these is true of white noise. More technically, movies constitute only a tiny fraction of the space of all possible activations of all the pixels on your screen. They (and indeed real visual scenes) have a particular statistical structure that the cortex is supposed to extract.

What is the cortex supposed to do with this structure? The idea is that the cortex learns to model, or ‘parameterize’, it. Then, the activities of cortical cells over time for a particular input, for example, a particular face in a movie, indicate the values of the parameters associated with that face. Thereby the cortical activities represent the input. The parameters for a face might include one set for its physical structure (e.g., the separation between the eyes and whether it is more round or more square), another set for the expression, and yet others, too.

Cortical representations are thus intended to reflect directly the statistical structure in the input. Importantly, for inputs such as movies, this structure is thought to be hierarchical and, concomitantly, to provide an account of the observed hierarchical structure of sensory cortical areas. One source of hierarchical structure in movies is the simple fact that objects (such as the faces) have parts (such as eyes and cheeks) whose form and changes in form over time are interdependent. Another source of hierarchical structure is that the same face can appear in many different poses, under many different forms of illumination, and so on. Pattern theory (Grenander 1995), one of the parent disciplines of the field, calls these dimensions of variation deformations. Loosely, the deformations are independent of the objects themselves, and we might expect this independence to be reflected in the cortical representations. Indeed, there is neurophysiological evidence for just such invariant neural responses to deformations of a stimulus.

How does the cortex do all this? Of course, some fraction of this structure was built in over evolution. However, the unsupervised learning tradition concentrates on ontogenic adaptation, based on multiple presented input movies. An additional facet of the lack of supervision is that this adaptation is taken as not depending on any particular behavioural task.

Finally, what does this process allow the cortex to do? The whole representational structure is intended to support inference. Crudely, this involves turning partial or noisy inputs into the completed, cleaned-up patterns they imply, using connections between areas in the cortical hierarchy. Construed this way, probabilistic inference actually instantiates a very general form of computation. Crucially, over the course of the development of unsupervised learning methods, it has been realised that the best way to approach the extraction of input structure, and inference with it, is through the language and tools of probability theory and statistics. The same realisation has driven substantial developments in artificial intelligence, machine learning, computer vision, and a host of other disciplines.

## Predictive Auto-Association

We can now return to the book. Hawkins compactly sums up his thesis in the following way. ‘To make predictions of future events, your neocortex has to store sequences of patterns. To recall appropriate memories, it has to retrieve patterns by their similarity to past patterns (auto-associative recall). And finally, memories have to be stored in an invariant form so that the knowledge of past events can be applied to new situations that are similar but not identical to the past.’In fact, to take the latter points first, the sort of auto-associative storage and recall to which Hawkins refers is a theoretically and practically hobbled version of unsupervised learning's probabilistic inference. Invariance is closely related to the deformations we described above in the context of pattern theory.

Unsupervised learning has certainly paid substantial attention to sequences of inputs and prediction, and to some good effect. For instance, (artificial) speech recognition programs are based on a probabilistic device called a hidden Markov model, which is a key element in a wealth of unsupervised learning approaches to prediction. However, despite heroic efforts, these modelling methods are incapable of capturing the sort of complex structure seen in inputs such as natural languages. They fail on phenomena like long-distance dependencies, for example, the agreement between the cases of subjects and verbs, which are rife. This does tend to offer a vaccine against Hawkins' otherwise infectious optimism.

Once place in which Hawkins goes beyond existing unsupervised learning models is in an extension to actions and control, and in an ascription of parts of the model to cortical anatomy. The hierarchical conception of cortex here goes all the way down to primary motor cortex (the neocortical area most directly associated with motor output). This allows auto-associative recall of sequences of past inputs and outputs to be used to specify actions that have formerly been successful. The discussion of this possibility is, unfortunately, rather brief. Central issues are omitted, such as the way that planning over multiple actions might happen. Also, the way that value is assigned to outcomes to determine success or failure is not discussed. The latter is widely believed to involve the neuromodulatory systems that lie below the cortex and that the book's cortical chauvinism leads it cheerfully to ignore.

By contrast, the book has a rather detailed description of how the model should map onto the anatomy of the cerebral cortex. Like many unsupervised learning modellers, Hawkins is a self-confessed ‘lumper’. He ignores huge swathes of complexity and specificity in cortical structure and connections in favour of a scheme of crystalline regularity. Though this will doubtless irk many readers (as will the lack of citations to some influential prior proponents such as Douglas and Martin [1991]), some (though not necessarily this) strong form of abstraction and omission is necessary to get to clear functional ideas. This part has interesting suggestions, such as a neat solution for a persistent dilemma for proponents of hierarchical models. The battle comes between cases in which information in a higher cortical area, acting as prior information, boosts activities in a lower cortical area, and cases of predictive coding, in which the higher cortical area informs the lower cortical area about what it already knows and therefore suppresses the information that the lower area would otherwise just repeat up the hierarchy. The proposed solution involves the invention (or rather prediction) of two different sorts of neurons in a particular layer of cortex.

Unsupervised learning models of cortex are without doubt very elegant. However, if pushed, purveyors of this approach will often admit to being kept awake at night by a number of critical concerns even apart from the difficulty of getting the models to work in interestingly rich sensory domains. Does the book provide computational Halcyon? First, the representations acquired by unsupervised learning are intended to be used for something-such as accomplishing more specific learning tasks, for example, making predictions of reward. However, most aspects of the statistical structure of inputs are irrelevant. This might be called the ‘carpet’problem: there is a wealth of statistical structure in the visual texture of carpets; however, this structure is irrelevant for almost any task. Capturing it might therefore (a) constitute a terrible waste of cortical representational power, or, worse, (b) interfere with, or warp, the parameterization of the aspects of the input that are important, making it harder to extract critical distinctions. The book does not address this issue, relying on there being enough predictive power to capture any and all predictions, including predictive characterisation of motor control.

Second, although our subjective sense is that we build a sophisticated predictive model of the entire sensory input, experiments into such phenomena as change blindness (Rensink 2002) show this probably isn't true. A classic example involves alternating the presentation of two pictures, which differ in some significant way (e.g., the colour of the trousers of one of the main protagonists). Subjects have great difficulty in identifying the difference between the pictures, even though (a) they are explicitly told to look for it, (b) they have the subjective sense that they have represented all the information in each picture, and (c) if the location of the change is pointed out, they see it as blindingly obvious. This, and other attentional phenomena, suggests that substantially less is actually represented than we might naively think. In fact, elaborate computations go into selecting aspects of the input to which the models might be applied, and sophisticated models of these computations, such as Li's salience circuit (2002), involve aspects of cortical anatomy and physiology ignored in the book.

As a final example of a spur to insomnia, unsupervised learners worry that Damasio (1994) might be somewhat right. That is, cool logic and hot emotion may be tightly coupled in a way that a model such as this that is rigidly confined to cortical processing, ignoring key subcortical contributions to practical decision making, will find hard to capture.

To sum up, in terms of the adage that genius is 1% inspiration and 99% perspiration, the book's enthymematic nature suggests that not quite enough sweat has been broken. Were it 1% inspiration and 99% aspiration, though, then the appealing call to arms for a new generation of modellers should more than suffice.

**Figure:**